# Immunolocalization of RANKL is Increased and OPG Decreased During Dietary Magnesium Deficiency in the Rat

**DOI:** 10.1186/1743-7075-2-24

**Published:** 2005-09-14

**Authors:** Robert K Rude, Helen E Gruber, Livia Y Wei, Angelica Frausto

**Affiliations:** 1 University of Southern California and the Orthopaedic Hospital, 1975 Zonal Ave., GNH 6602, Los Angeles, CA 90089-9317, USA; 2 Department of Orthopaedic Surgery, Carolinas Medical Center, P.O. Box 32861, Charlotte, NC 28203, USA

## Abstract

**Background:**

Epidemiological studies have linked low dietary magnesium (Mg) to low bone mineral density and osteoporosis. Mg deficiency in animal models has demonstrated a reduction in bone mass and increase in skeletal fragility. One major mechanism appears to be an increase in osteoclast number and bone resorption. The final pathway of osteoclastogenesis involves three constituents of a cytokine system: receptor activator of nuclear factor kB ligand (RANKL); its receptor, receptor activator of nuclear factor kB (RANK); and its soluble decoy receptor, osteoprotegerin (OPG). The relative presence of RANKL and OPG dictates osteoclastogenesis. The objective of this study was to assess the presence of RANKL and OPG in rats on a low Mg diet.

**Methods:**

RANKL and OPG were assessed by immunocytochemistry staining in the tibia for up to 6 months in control rats on regular Mg intake (0.5 g/kg) and experimental rats on reduction of dietary Mg (.04%, 25% and 50% of this Nutrient Requirement).

**Results:**

At all dietary Mg intakes, alteration in the presence of immunocytochemical staining of RANKL and OPG was observed. In general, OPG was decreased and RANKL increased, reflecting an alteration in the RANKL/OPG ratio toward increased osteoclastogenesis.

**Conclusion:**

We have, for the first time demonstrated that a reduction in dietary Mg in the rat alters the presence of RANKL and OPG and may explain the increase in osteoclast number and decrease in bone mass in this animal model. As some of these dietary intake reductions in terms of the RDA are present in a large segment of or population, Mg deficiency may be another risk factor for osteoporosis.

## Introduction

Severe magnesium (Mg) deficiency (0.04% of nutrient requirement, NR)[1] results in osteoporosis in rodent models characterized by decreased bone formation, increased bone resorption, and increased skeletal fragility [2-8]. This degree of Mg depletion rarely exists in humans; however, we have also found that a more moderate dietary Mg restriction, 10% of NR and 25% of NR, also results in bone loss in the rat [9,10]. We have also recently found that a diet of 50% of NR also causes a reduction in bone mass (submitted for publication). These studies suggest that an inadequate Mg intake may be a risk for osteoporosis. The RDA for Mg for adult males is 420 mg/d and for adult females is 320 mg/d [11,12]; the usual dietary Mg intake however falls below this recommendation in a large proportion of the population [11-13]. Epidemiologic studies have demonstrated a positive correlation between dietary Mg intake, and bone density and/or an increased rate of bone loss with low dietary Mg intake suggesting that dietary Mg deficiency may be a risk factor for osteoporosis [13-18].

The cause of this effect of Mg depletion on bone is unclear, although increased amounts of inflammatory cytokines have been found in bone of Mg deficient mice and rats which could increase bone resorption [7,9,10]. The final pathway of osteoclastogenesis has been proposed to involve three constituents of a cytokine system: receptor activator of nuclear factor kB ligand (RANKL); its receptor, receptor activator of nuclear factor kB (RANK); and its soluble decoy receptor, osteoprotegerin (OPG) [19,20]. RANKL is a membrane bound cytokine-like molecule that is expressed in preosteoblastic cells. It stimulates the differentiation, survival, and fusion of osteoclastic precursor cells to activate mature RANK expressed in hematopoietic osteoclast progenitors, and serves as an essential factor for osteoclastic differentiation and activation. RANKL binds to RANK with high affinity and this interaction essential for osteoclastogenesis. OPG is expressed in a variety of cell types, however in bone it is mainly produced by cells of osteoblastic lineage. OPG has very potent inhibitory effects on osteoclast formation. It acts like a decoy receptor and blocks the RANKL/ RANK interaction [19]. The relative presence of RANKL and OPG therefore dictates osteoclast bone resorption activity [21-23]. It was the objective of this study to examine the effect of reduction in dietary Mg on immunocytochemical presence of RANKL and OPG in Mg deficient vs. control animals.

## Material and methods

### Experimental Methods

Studies reported here were approved by the IACUC at the University of Southern California. Dietary Mg deficiency was induced for up to 6 months in 6 week old, 150–175 g female Sprague Dawley rats (Charles Rivers Laboratory, Wilmington, MA). After acclimation to the vivarium as previously described [9,10], experimental diets were instituted. Group pair feeding based on food weight was performed daily in order to keep weight gain as close as possible in the Mg deficient and control groups as bone mass is closely correlated with body mass. Distilled deionized water containing < 3 × 10^-5 ^g Mg/L was used for hydration. Rats were fed either a normal control Mg diet of 0.05 % Mg (0.5 g/kg or 100% of NR as percent of total diet) or a Mg-deficient diet (0.04%, 25%, and 50% of NR Mg) (Harlan Teklad, Madison, WI) as previously described [10]. The dietary intake of calcium (Ca) was at or near the recommended intake for rats at 0.5 %.

At the end of each experimental period, rats were anesthetized with ketamine, 50 mg/kg, and zylazine, 10 mg/kg, intramuscularly (Phoenix Pharmaceuticals Inc, St. Joseph, MO). Blood samples from the anesthetized rats were obtained by cardiac puncture and rats were then killed by open thoracotomy. The femurs and tibias were harvested at each time point for mineral analysis, micro-computerized tomography, histomorphometry, and immunocytochemistry. Prior publications review the results in terms of serology, bone mineral density and content and the presence of inflammatory cytokines [7,9,10].

### Immunocytochemical Localizations of RANKL and OPG

The tibia was isolated immediately following euthanasia and fixed in 2% NBF (Neutral Buffered Formalin) for 24 hrs and decalcified in 20% EDTA 0.1 M Tris pH 7.2–7.3 solution. After dehydration and paraffin embedding at 56°C, sections were cut so that a sagittal section including the epiphysis and the metaphysis of the tibia was obtained. As previously described, indirect immunocytochemistry [7,9,10] was used to localize RANKL and OPG; both primary and secondary spongiosa were evaluated as described below. RANKL and OPG antibodies were obtained from R&D Systems, Minneapolis, MN. The source of antibody was goat. Biotin labeled goat antibody as the second antibody and streptavidin-HRP attached to the antibody complex completed immunolocalization. Rat small intestine tissue containing Peyer's patches and spleen sections served as a positive control for both RANKL and OPG antibodies. If the result was comparable with the established positive staining using the same experimental conditions, the procedure was validated.

Localization was visualized in the light microscope using peroxidase substrate containing red dye Nova Red (Vector Labs, Burlingame, CA) and counterstained with Hematoxylin (blue)(Zymed Laboratories, South San Francisco, CA). The results were photographed in a Zeiss photo microscope (Carl Zeiss, Thornwood, NY, USA) using a 40 X objective.

### Evaluation of Cytokine Localizations

Background localization was minimal compared with positive and negative controls. No difference in background localization was observed between Mg deficient and control rats. Cells and tissues stained specifically as described for the antigen in the literature [24,25]. Intensity was graded as 0 = no localization, 1 = weak localization, 2 = moderate, and 3 = strong localization. The quantitative estimate of numbers of cells staining was a = <20%, b = 20–60%, and c = >60%. The mean relative positivity was <1b = 0; 2a and 2b = 1; and 3b and 3c = 2 [24,25]. In this study, in some instances, no clear staining was observed for OPG at 4 and 6 months in animals on the 25% NR Mg diet, therefore, a minimum value of 0.01 was given to allow for calculation of RANKL/OPG ratio.

## Results

**Figure 1:**
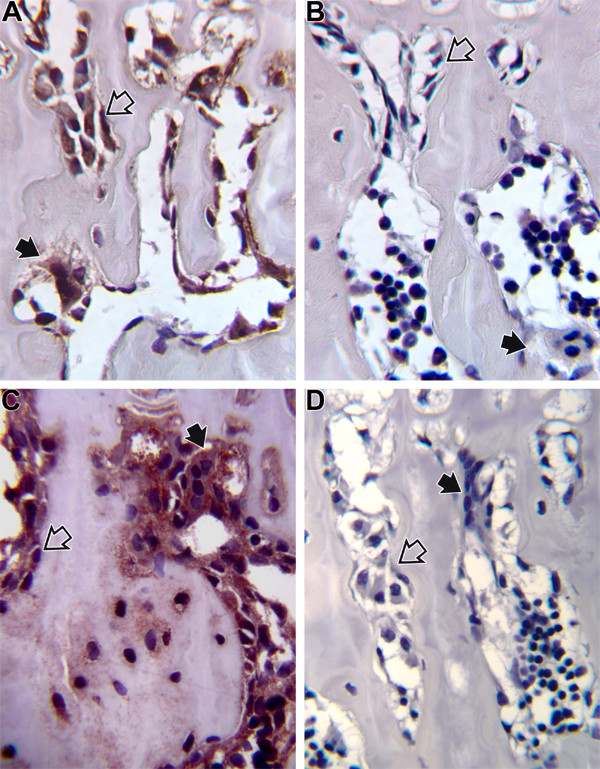
Immunocytochemical staining of RANKL in bone from Mg deficient and control animals. A. Represents animals on a 25% NR diet and B. the control group. Note the positive staining of osteoclasts (solid arrows) and osteoblasts (open arrows) in A. while minimal staining is observed the control animals (B). C. Represents animals on a 50% NR diet and D. the control group. Again, as observed in C., Mg deficient animals have much more intense staining of RANKL of osteoclasts (solid arrows) and osteoblasts (open arrows) than is observed the control animals (D).

**Figure 2:**
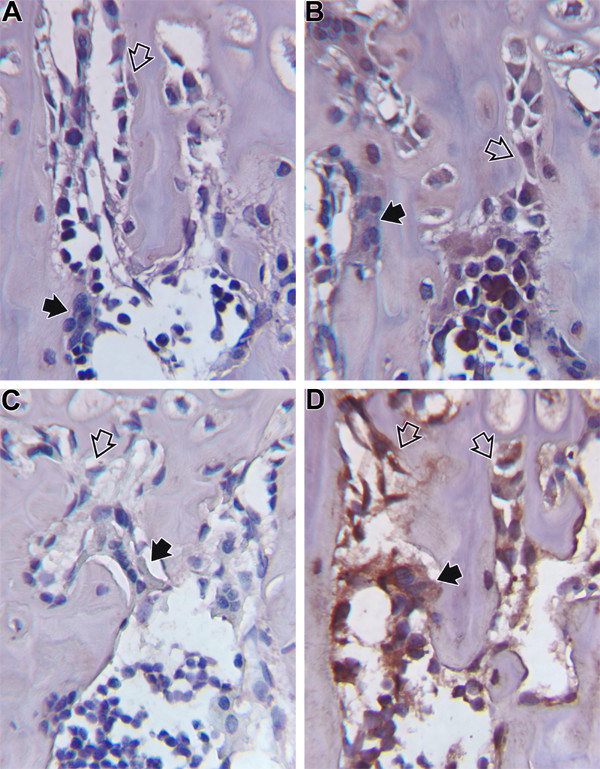
Immunocytochemical staining of OPG in bone from Mg deficient and control animals. A. Represents animals on a 25% NR diet and B. the control group. Note the minimal staining of osteoclasts (solid arrows) and osteoblasts (open arrows) in A. while positive staining is observed the control animals (B). C. Represents animals on a 50% NR diet and D. the control group. Again, as observed in C., Mg deficient animals have minimal staining for OPG in osteoclasts (solid arrows) and osteoblasts (open arrows), while much more intense staining is observed in the control animals (D).

Our prior studies have documented that dietary Mg intake at .04% 10%, and 25% of NR in the rat results in bone loss and an increase in inflammatory cytokines TNFα and IL1β [9,10]. Similar observations have been made at the 50% NR level (submitted for publication). As discussed in the Introduction, the final pathway of osteoclastogenesis has been proposed to involve RANKL and OPG [19-23]. We have examined the immunocytochemical presence of these two cytokines in Mg deficient vs. control animals. Examples of immunocytochemical staining are shown for RANKL in Figure 1 and for OPG in Figure 2. In rats fed a diet containing 0.4% of NR, there was an increase in staining in many cells of the bone microenvironment; RANKL was increased by 448% in osteoblasts on day 1, in lymphocytes by 157% on day 3 and 100% on day 8, and in osteoclasts by 64% on day 22. No difference was observed at 28 or 84 days of depletion. OPG, in contrast, was reduced in osteoblasts by 75% on day 3 and 149% by day 21. In mononuclear cells, OPG was reduced by 53% on day 1, 100% on day 3, and 233 % on day 8. This effect continued by day 28 when OPG was decreased in osteoblasts by 27% and in osteoclasts by 74%. At 12 weeks of Mg depletion OPG had decreased in all components of the bone environment: osteoblasts by 108%, osteoclasts by 167%, megakaryocytes by 85%, and mononuclear cells by 92%. While these percentage changes are of interest, it is the relative presence of RANKL to OPG in bone that dictates overall effect on osteoclastogenesis. The ratio of RANKL/OPG relative immunostaining was calculated at this dietary intake and is shown in Table 1; the higher the value the greater the preponderance of RANKL. As noted, it was only on or after day 22 of the experimental diet that the ratio data suggest an excess of RANKL.

**Table 1:** Ratio of RANKL/OPG in Osteoclasts and Osteoblasts in Rats on a .04% NR Diet

	Osteoclast	Osteoblast
Day 1 Control	1	0.44
Day 1 .04% NR	0.4	.046
Day 3 Control	0.6	0.55
Day 3 .04% NR	0.51	0.24
Day 8 Control	1	0.44
Day 8 .04% NR	0.25	0.33
Day 15 Control	1.25	0.29
Day 15 .04% NR	0.79	0.38
Day 22 Control	0.65	0.05
Day 22 .04% NR	0.12	0.25
Day 28 Control	.029	0.12
Day 28 .04% NR	.052	0.16
Day 84 Control	.096	0.14
Day 84 .04% NR	0.256	1.66

As shown in Table 1 there is no apparent excess of RANKL relative to OPG in rats on a diet containing .04% of NR until after day 22.

Samples were obtained at 2, 4, and 6 months from animals on the 25% NR intake. The percent changes and ratio of RANKL/OPG are shown in Tables 2 and 3. RANKL appears higher at the 2 and 6 month points in osteoclasts, but is lower in osteoblasts from animals with Mg deficiency. OPG was quite suppressed in both ostoclasts and osteoblasts in Mg deficient animals. The ratio calculated, however, showed a preponderance of RANKL to OPG.

**Table 2:** Percent Difference in Immunocytochemical Staining for RANKL and OPG

	25% NR			
	Osteoclast		Osteoblast	

	RANKL	OPG	RANKL	OPG

2 Month 25% vs Control	86	-150	-10	-113
4 Month 25% vs Control	-16	-160	-232	-160
6 Month 25% vs Control	116	-700	-82	-700

As shown in Table 2, the percent relative difference in immunocytochemical staining in rats on a diet containing 25% NR Mg for RANKL in osteoclasts was increased at 2 and 6 months and decreased in osteoblasts. Staining for OPG was markedly decreased in both cell lines at all time points compared to control.

At the 50% NR dietary intake, samples were obtained at 3 and 6 months. These data are shown in Tables 4 and 5. At this dietary intake level, RANKL is increased in both osteoclasts and osteoblasts of Mg depleted rats while OPG is decreased. The ratios of RANKL/OPG again favor a relative increase in RANKL in Mg deficiency.

**Table 3:** Ratio of RANKL/OPG in Osteoclasts and Osteoblasts in Rats on 25% NR Mg Diet

	25% NR	
	Osteoclast	Osteoblast

2 Month Control	2.32	9.82
2 Month 25% NR	108	18.75
4 Month Control	3.41	4.88
4 Month 25% NR	50	25
6 Month Control	3.12	2.12
6 Month 25% NR	54	31

As shown in Table 3, the ratio of RANKL to OPG was increased in Mg deficient animals relative to controls in both cell types which favors osteoclastogenesis.

**Table 4:** Percent Difference in Immunocytochemical Staining for RANKL and OPG

	50% NR			
	Osteoclast		Osteoblast	

	RANKL	OPG	RANKL	OPG

3 Month 50% vs Control	+250	-152	+320	-20
6 Month 50% vs Control	+240	0	+700	-100

As shown in Table 4, the percent relative difference in immunocytochemical staining in rats on a diet containing 50% NR Mg for RANKL was increased at 3 and 6 months in osteoclasts and in osteoblasts. Staining for OPG was decreased in both cell lines three of the four time points compared to control.

**Table 5:** Ratio of RANKL/OPG in Osteoclasts and Osteoblasts in Rats on 50% NR Mg Diet

	50% NR	
	Osteoclast	Osteoblast

3 Month Control	0.24	0.12
3 Month 25% NR	0.30	0.42
6 Month Control	0.13	0.02
6 Month 50% NR	3.13	0.32

As shown in Table 5, the ratio of RANKL to OPG was increased in Mg deficient animals relative to controls in both cell types which favors osteoclastogenesis.

## Discussion

In our prior publications, we have clearly demonstrated that Mg deficiency results in bone loss and is accompanied by an increase in osteoclast number and indices of bone resorption [6-10]. It was hypothesized that since Mg depletion induces a rise in substance P with subsequent stimulation of inflammatory cytokines such and TNFα, IL1β, and IL6, that this may be the mechanism for bone loss [26]. Indeed, we have demonstrated an increase in the immunolocalization of TNFα and IL1β in the Mg deficient rat and mouse [7,9,10]. Both cytokines are known to stimulate osteoclastic bone resorption. These changes were observed to begin very early in the course of Mg depletion. In contrast, the relative change in RANKL to OPG to favor bone resorption did not occur until at least 4–6 weeks into depletion at the dietary intake of .04% NR. The relative presence of RANKL and OPG dictates osteoclast bone resorption activity as discussed above. Osteoclasts can be formed or activated in a RANKL and/or a RANKL-independent mechanism by TNFα [19-23]; therefore, TNFα and IL1β may be directly responsible for the early osteoclastic bone resorption and the decline in OPG and an increase in RANKL/OPG which follows later in the course of depletion. Responses to varying dietary intakes also appear to differ in absolute changes in RANKL and OPG. We did not assess early changes at the 25% or 50% NR, and thus we do not known if there were any changes in RANKL or OPG at these time points with these higher Mg diets. As was observed at the 3 months time point (84 days) in the .04% NR diet, there was a remarkable fall in OPG at the higher Mg diets relative to control. While RANKL was also increased in osteoclasts at both the 25% and 50% NR diets, an increase was only observed in osteoblasts at the 50% NR diet. The reason for this is unclear. The ratio of RANKL/OPG throughout favors osteoclastogenesis and suggests that this may be a major mechanism for the increase bone loss in Mg deficiency.

It is clear that dietary Mg deprivation does result in a reduction in bone mass and that there may be other reasons for decrease bone mass as well. We have also observed a decrease in osteoblastic bone formation [6,7,9,10]. This could be related to a decrease in PTH and 1,25(OH)_2_-vitamin D relative to control [9,10,27,28]. Several other potential mechanisms may account for a decrease in bone mass/strength during Mg deficiency. Mg is mitogenic for bone cell growth, and therefore Mg deficiency may result in a decrease in bone formation [29]. Mg also affects crystal formation; a lack of Mg results in a larger, more perfect crystal which may affect bone strength [30]. Serum IGF-1 levels have also been observed to be low in the Mg deficient rat; decreased IGF-1 may adversely influence skeletal growth[31].

A limitation of studies employing immunocytochemistry is that this approach may be influenced by observer subjectivity and bias and intra-observer variation. Our findings, however, are provocative and further assessment employing other techniques are indicated and may include assessment of cytokine gene expression using both in situ and laser capture microdissection/gene array analyses. These approaches would address the question of whether low Mg intake influences gene cytokine expression, and allow study of specific cell types such as mononuclear cells, osteoblasts and osteoclasts. We have, by in situ hybridization data, demonstrated much greater gene expression of substance P and of TNFα in rats on a 10% NR Mg diet compared to control animals (unpublished data).

## Conclusion

We have, for the first time, demonstrated that a reduction in dietary Mg in the rat alters the presence of RANKL and OPG and may explain the increase in osteoclast number and decrease in bone mass in this animal model. As these dietary intake reductions in terms of the RDA are present in a large segment of or population, Mg deficiency may be another risk factor for osteoporosis.

## Competing interests

The author(s) declare that they have no competing interests.

## Authors' contributions

RKR designed the study and supervised the experimental work, analyzed data, and drafted the manuscript. HEG assisted in the study design and interpretation of the data. LYW was responsible for the care and pair feeding of the animals and interaction with the veterinarian. AF performed the immunocytochemical studies and generated images as well as preparing the bone for these experiments.
